# Enhancing Student Interest in Animals. Commentary: A Crisis in Comparative Psychology: Where Have All the Undergraduates Gone?

**DOI:** 10.3389/fpsyg.2015.01897

**Published:** 2015-12-17

**Authors:** Carla Krachun

**Affiliations:** Psychology, Memorial University of Newfoundland, Grenfell CampusCorner Brook, NL, Canada

**Keywords:** comparative psychology, recruitment, undergraduates, student perceptions, career options

Abramson's (2015) article confirmed what I have come to realize in several years of teaching: many psychology students have a general lack of enthusiasm for studying animals. In my department, the consequence is that courses in animal behavior and cognition have been offered on an infrequent basis, canceled due to low enrollment, or given more human content to widen their appeal. For example, a third-year course on learning processes that traditionally focused on animal research now covers learning disabilities or language learning.

Abramson suggests that a number of factors contribute to our difficulties with recruiting students in comparative psychology, including a paucity of courses and graduate programs, a lack of choice in textbooks, and sparse mention of the subject in introductory texts. Domjan and Purdy (1995) also reported a common failure of introductory texts to acknowledge animal studies, and they found that findings from animal research were often presented as if they had been obtained from humans. All of this sends students the message that research with animals is uncommon, unimportant, or irrelevant in psychology.

Another way we send this message is in our failure to portray the animal aspect of psychology on university websites. Most undergraduate program homepages include a brief definition of psychology and/or a description of what psychologists do. I work in Canada, so I took a look at the (non-exhaustive) list of universities on the Canadian Psychological Association's website^1^. Of 55 institutions offering undergraduate programs, less than a third referred to animals (including using words like “organisms” or “other species”) in their descriptions of psychology. Most websites also included an image on the undergraduate homepage, depicting everything from inkblots, to brain scans, to students and faculty engaged in research. However, in only a couple of instances did the image include or make reference to animals. Granted, departments without animal research facilities may not want to mislead prospective students; but they do not need to have such facilities to acknowledge that psychology, as a field, involves the study of both human *and* nonhuman animals. Abramson mentions that introductory textbooks are students' first source of information about a field, but students may form even earlier impressions while perusing university websites. While most of us have little say in the content of introductory textbooks, we can all quickly and easily check our program website to make sure it acknowledges the study of animals in psychology. If yours does not, you can contact the person responsible for website content to request that this be corrected.

Students might also be more interested in learning about animals if they recognized it as relevant to their career goals. Abramson suggests teaching students about the value of comparative psychology for developing broad skills widely sought after by employers. Making students aware of the many exciting animal-related professions that exist could also help in recruiting more students into animal courses. A quick internet search returned careers such as pet adoption counselor, humane educator, zoo habitat designer, service animal trainer, animal welfare officer, animal legislation lawyer, wildlife rehabilitator, sanctuary manager, conservation fundraiser, and animal-assisted therapist, among many others. Cornell University has a fantastic website^2^ listing over 180 professions involving animals. I am not endorsing the corporate model whereby a university education is tailored to prepare students for specific employment. However, students often do choose courses at least partly based on how relevant they are to their chosen careers (e.g., students planning to work with children are interested in developmental psychology courses). While we should encourage—perhaps even require—undergraduates to take courses in comparative psychology for a well-rounded education, there is nothing wrong with also making them aware of careers that are well-suited for psychology graduates with an interest in animals. My institution employs a career planning coordinator who gives presentations to introductory classes on employment options for psychology graduates. If your institution has a similar person on staff, you could ask him/her to include information on animal-related careers.

Finally, the lack of enthusiasm many students have for studying animals may be based on incomplete or erroneous information about the role of animals in psychology. Some students disagree on moral/ethical grounds with causing pain or discomfort to animals for teaching or research (Cunningham, 2000). However, they may be unaware that a great number of animal studies in psychology consist of noninvasive behavioral research (see Beran et al., 2014). As Abramson suggests, students may also not fully appreciate the extent to which research with animals offers important insights into human behavior. This comes back to the shortage of courses in comparative psychology, where students would normally obtain this information. Abramson proposes several ways to reach students outside of formal courses, such as giving presentations at high schools or special events. Another way to reach students within your own institution is to give guest lectures in colleagues' classes on relevant topics in comparative psychology. For example, research on personality in animals could be presented as a way of gaining insight into human personality (Gosling, 2001). Colleagues are usually more than happy to welcome a guest lecturer, so this may be a way to reach undergraduates in introductory courses when you do not teach those courses yourself.

The ideas presented here are admittedly small measures. Other approaches that have the potential to make a greater impact should, of course, also be pursued. These include, for example, recruiting students into our labs where they can experience animal research first-hand; encouraging the hiring of colleagues who have an interest in comparative psychology; and pushing to have more animal courses offered or cross-listed with other programs. Anyone who is in a position to do these things should definitely do them, and most of us likely already are. However, some people (e.g., contractual or sessional faculty) may not have the resources or influence to take such measures. What I have done here is to suggest three tangible and easy actions that any one of us can take to help get more students more interested in animals.

## Conflict of interest statement

The author declares that the research was conducted in the absence of any commercial or financial relationships that could be construed as a potential conflict of interest.

## Footnotes

^1^http://www.cpa.ca/students/resources/canadianuniversities.

^2^http://animalcareers.cornell.edu/.
